# Drugs, diet, or surgery? How treatment type and willpower shape responsibility for weight loss and judgments of the physician and treatment

**DOI:** 10.3389/fpsyg.2026.1772798

**Published:** 2026-04-22

**Authors:** Serena Petrocchi, Giulia Biaggini, Nicolò Zallocco

**Affiliations:** Institute of Family Medicine, Università della Svizzera italiana, Lugano, Switzerland

**Keywords:** doctor-patient relationship, GLP-1, obesity stigma, treatment judgments, trust, weight-loss treatments, willpower

## Abstract

**Introduction:**

Grounded in attribution theory and informed by the perspective on health moralization, this study examined how perceived lack of willpower shapes individuals’ judgments of responsibility for weight loss in individuals with obesity across different treatment methods (diet/exercise, bariatric surgery, and weight loss drugs). The study also investigated how willpower attributions and treatment type shape evaluations of the doctor, including trust, perceived credibility, and treatment acceptance.

**Methods and measures:**

This study used an experimental vignette design in which the weight loss method (diet/exercise vs. bariatric surgery vs. weight loss drugs) was experimentally manipulated, and perceived lack of willpower was measured. The study included 183 adult participants (*M* age = 29.3, SD = 10.98; 133 women).

**Results:**

The moderation analyses revealed that perceived lack of willpower predicted lower responsibility for weight loss, but this effect was conditional on treatment type and emerged primarily in the weight loss drug condition. Across all conditions, treatment type had a robust effect: pharmacological treatments were consistently associated with lower perceived responsibility, lower trust in the doctor, lower perceived credibility, and lower treatment acceptance than surgery and diet/exercise. Perceived lack of willpower did not influence evaluations of the doctor or treatment.

**Conclusion:**

These findings suggest that individuals rely on distinct judgment processes when evaluating both patients and treatment providers: responsibility attributions are shaped by dispositional beliefs about willpower, whereas evaluations of doctors and treatments are driven by treatment-related cues of effort and legitimacy. Understanding how treatment framing and beliefs about willpower interact may help clinicians communicate more effectively and foster trust in weight management interventions.

## Introduction

Weight-related stigma remains pervasive across multiple social contexts, including healthcare settings. Individuals with high weight often face negative biases even in doctor–patient relationships (Alberga et al., 2018; Phelan et al., 2015; Puhl et al., 2021; Ryan et al., 2024), where stigma may negatively lead to decreased trust, lower perceived empathy, and reduced treatment adherence (Gudzune et al., 2014). A key factor in weight stigma is how people attribute the causes of obesity and evaluate attempts to manage it. Attribution theory (Fiske and Taylor, 1991) suggests that causal judgments are shaped by three key dimensions: locus (whether the cause is internal or external to the person), stability (whether the cause is perceived as enduring or temporary), and controllability (whether the individual is seen as able to influence the outcome) (Sullivan, 1975). When obesity is attributed to internal, relatively stable, and controllable factors, such as a lack of self-control or willpower (Westbury et al., 2023; Carels et al., 2009), the emphasis falls on personal discipline and control (Crandall and Cohen, 1994). This reinforces negative stereotypes, perpetuates stigma (Crandall and Schiffhauer, 1998), and anti-fat attitudes (Crandall, 1994; Crandall and Schiffhauer, 1998). Consistent with this view, research has shown that individuals with obesity perceived as lacking willpower are described as lazy and lonely (Crandall and Martinez, 1996). Others have demonstrated that a free will beliefs-based mindset regarding individuals with obesity is correlated with blaming toward them (Chandrashekar, 2020).

Another perspective aligning with these attributional processes, while providing additional insights, is health moralization (Rozin and Singh, 1999). When a neutral behavior is moralized, it acquires either a positive or negative ethical value and becomes more significant than others (Feinberg et al., 2019). In this vein, obesity is often viewed not as the result of complex biological, social, and environmental influences (Puhl and Brownell, 2003) but rather as a reflection of personal failure or moral weakness (Oswald, 2024). This perspective labels individuals who are overweight as irresponsible, undisciplined, and morally failing to manage food and physical activity properly (Crandall and Schiffhauer, 1998; Puhl and Heuer, 2009). Taken together, the attribution theory and the perspectives on health moralization provide a useful framework for understanding how perceptions of obesity may be shaped by beliefs about personal control and individual responsibility.

Building on this reasoning, attributing obesity to a lack of willpower implies that weight gain results from the individual’s failure to exert control. However, this same attribution may also shape how weight loss is interpreted. If individuals are perceived as lacking willpower, successful weight loss may be less likely to be attributed to their own effort and self-discipline, and more likely to be seen as driven by external factors or circumstances. As a result, individuals with obesity may be assigned less personal responsibility for weight loss when their condition is attributed to a lack of willpower.

The belief that obesity can be easily addressed by simply eating less and exercising more leads to the marginalization of treatments beyond lifestyle interventions. As a result, individuals who seek medical treatment for obesity may be stigmatized, as they are not seen as responsible for their weight loss and are deemed undeserving of such care. For example, Mattingly et al. (2009) found that the weight loss method influences how responsible individuals are perceived for their weight loss, with diet being perceived as more effortful than bariatric surgery. Bullock et al. (2011) demonstrated that the combination of anti-fat attitudes and perceived high weight-loss effort positively influenced perceptions of the sociability of individuals with obesity. Vartanian and Fardouly (2013) and Vartanian and Fardouly (2014) found that individuals who lost weight through diet or a combination of diet and surgery were thought to have greater control over their weight loss than those who lost weight solely through surgery. Additionally, obese individuals in the surgery condition were perceived as less pleasant and competent. Greater moralization was associated with negative responses toward individuals who underwent bariatric surgery, even when they made significant efforts with diet and exercise to maintain their weight loss (Ringel and Ditto, 2019). While these studies have primarily focused on surgical and dietary interventions, there has been relatively little empirical investigation into perceptions of pharmaceutical treatments for weight loss (e.g., GLP-1). Given the increasing availability and visibility of weight loss drugs in public discourse (Harjunen and Puhakka, 2025), it is critical to understand how pharmaceutical treatments are evaluated (see the preprint by Bachmakova et al., 2025). This study seeks to fill this gap by examining how perceived willpower and treatment method (diet/exercise, bariatric surgery, or weight loss drugs) jointly shape responsibility attributions for weight loss.

Beyond understanding how individuals attribute responsibility for weight loss, it is crucial to explore whether attribution processes affect the doctor–patient relationship and influence patients’ judgments of their healthcare providers and treatments. Prior evidence suggests that when patients with obesity perceive stigmatizing attitudes from healthcare providers, they are less likely to engage in weight loss attempts (Gudzune et al., 2014). Moreover, strong negative attitudes toward obesity by healthcare providers impact the care they provide and are the basis for stress, avoidant behaviors, and low trust in their patients (Phelan et al., 2015). In a different context, Haywood et al. (2014) found that patients with sickle cell disease who perceived discrimination from healthcare providers reported lower trust, which was associated with decreased adherence to medical recommendations. Taken together, these findings suggest that attribution processes in the clinical encounter can harm the doctor–patient relationship and affect treatment acceptance.

Extending this to the context of obesity, when certain weight loss treatments are viewed as morally questionable or effort avoidant (Grannell et al., 2021), they may also undermine trust in the physician, reduce acceptance of the recommended treatment, and affect judgments of the doctor’s competence. Since there is no prior data to support a directional hypothesis, this study seeks to fill this gap by examining whether perceptions of patient willpower across different types of weight loss treatments jointly shape evaluations of the doctor–patient relationship.

### Overview of the research

This study used an experimental between-subjects design in which the weight loss method (drugs vs. bariatric surgery vs. diet/exercise) was manipulated through narrative vignettes, and the perceived lack of willpower was measured. The study hypothesized that:

HP1: Building on prior research conceptualizing willpower as an internal, stable, and relatively controllable factor, we expected that a higher perceived lack of willpower would lead individuals with obesity to be seen as less personally responsible for weight loss.

HP2: Based on partial prior evidence, we expected that treatment methods perceived as more externally driven and less controllable (i.e., prescribed drugs or bariatric surgery) would lead participants to attribute less responsibility for weight loss to the individual than more internally controlled methods (diet and exercise).

HP3: The study further expected that the effect of a high perceived lack of willpower on responsibility judgments would be amplified under treatment conditions emphasizing lower controllability and an external locus of control (drugs and surgery), compared to those emphasizing higher controllability and an internal locus of control (diet and exercise).

HP4. This study tested the main effect of a lack of willpower on participants’ evaluations of the doctor–patient relationship as part of the analysis. However, this study did not expect to find a significant effect since the doctor–patient relationship is shaped by other factors, such as the doctor’s professionalism, communication skills, and the patient’s trust in healthcare professionals.

HP5. This study hypothesized that the type of weight loss treatment would influence participants’ evaluations of the doctor–patient relationship. Specifically, treatments perceived as less controllable and externally regulated (e.g., weight loss drugs) would elicit more negative evaluations than those perceived as more effortful, controllable, and stable (e.g., bariatric surgery or diet/exercise). This expectation is grounded in the attributional view that when treatments are framed as “quick fixes” with lower personal involvement, observers attribute less responsibility and self-discipline to the patient and therefore evaluate the doctor less favorably for recommending such treatments.

RQ1: Finally, this study formulated a research question: What is the effect of the interaction between perceived lack of willpower and weight loss treatment type on participants’ evaluations of the judgment of the physician and the treatment?

## Methods

### Procedure

Participants answered questions about socio-demographic variables and willpower first, and then were randomized to one of the three experimental conditions. The conditions were based on vignettes depicting a consultation between a general practitioner (GP) and a patient (the character’s gender matched the participant’s gender). The story explained that a doctor addressed a patient’s condition of obesity and recommended either a pharmacological treatment using anti-diabetic medications, bariatric surgery, or diet/exercise. After that, the participants answered questions about the character’s responsibility for weight loss and the doctor’s evaluation of the story. The vignettes were pre-tested in a pilot study with 15 participants, which established that they were realistic and clear, and that participants had a sufficient understanding of anti-diabetic medications and bariatric surgery.

The University Ethical Committee approved the research project (CE decision 2024_01); the research was conducted in accordance with the principles of the Declaration of Helsinki. Participants did not receive compensation and answered anonymous questionnaires online, through Qualtrics™ links spread across several social media platforms and through word of mouth. Data collection took place from June 2024 to January 2025 in Switzerland.

### Participants

An *a priori* power analysis with ANCOVA (fixed/main/interaction effects), a prudential partial *ƞ*^2^ = 0.05, a power of 0.80, reported a *N* of 187. Participants were 183 adults (133 females) aged 18 to 72 (*M* = 29.30, SD = 10.98). Based on self-reported height and weight, mean BMI indicated a condition of overweight for males and normal weight for females (males *M* = 26.1, SD = 2.9, range 18–32; females *M* = 22.5, SD = 4.7, range 15–53). In addition, participants reported their subjective perception of their weight status: 20% considered themselves normal weight or underweight, whereas 79% perceived themselves as overweight or obese.

Regarding socio-demographic variables, 52% of the sample had completed high school or acquired an equivalent diploma, and 47% had a university degree. Most participants (56%) had no source of income, and 60% were in a stable relationship. 10% of participants have a chronic disease, and 71% rate their health status as good/optimal. 55% followed a diet at least once in their life to lose weight. 45% had a family member or a friend suffering from obesity. According to the randomization, 58 participants were assigned to the weight loss drug condition, 61 to the diet/exercise condition, and 64 to the surgery condition.

### Data analysis strategy

Data analyses of the three studies were conducted in SPSS v.29. The data were screened for missing values and univariate distributions. Omega’s *ω* was used to evaluate the reliability of the scales. To test the hypotheses and the research questions, moderation analyses were conducted using regression models (PROCESS; Hayes, 2022). Perceived lack of willpower was treated as a continuous predictor, and weight loss methods (diet/exercise vs. weight loss drugs vs. bariatric surgery) were entered as a categorical moderator. Interaction terms were included to examine whether the effect of perceived lack of willpower on the dependent variables varied across treatment conditions. Relevant covariates (e.g., gender, BMI, and dieting status) were included in the models. Significant interactions were further examined using conditional effect analyses, and 95% bootstrap confidence intervals were estimated from 5,000 resamples. Before conducting the main analyses, additional analyses tested for potential non-linear effects of perceived lack of willpower on the outcomes. No significant non-linear effects emerged, supporting the assumption of linearity.

### Measures

*Lack of Willpower* was measured with the 7-item of the Anti-Fat Attitudes scale (Crandall, 1994). This subscale captures both causal attributions and beliefs related to personal responsibility and blame. Examples of items are: people who weigh too much could lose at least some part of their weight through a little exercise (reverse); some people are fat because they have no willpower; and fat people tend to be fat pretty much through their own fault. Every item was used on a 5-point Likert scale (1 = completely disagree and 5 = totally agree). The items were averaged to create a final score, with higher scores reflecting a greater attribution of lack of willpower to individuals with obesity (*ω* = 0.87, rs > 0.74).

*Manipulation exposure*. The three vignettes were derived and adapted from previous research (Vartanian and Fardouly, 2014) in two versions, one for males and one for females, to match the participants’ gender to enhance ecological validity, facilitate perspective-taking, and minimize potential bias related to gender stereotypes. An example of the story is: “Doctor Brown is visiting his patient Lucy (or Mark), a 42-year-old lady (or man) who weighs 110 Kg and is 1.65 mt tall (or 123 Kg and 1.85 mt tall). Dr. Brown explains to Lucy that she is obese and recommends starting a pharmacological treatment with a drug developed for diabetes. These drugs have been found to be effective for weight loss and for avoiding the negative consequences that obesity can have on her health. Lucy follows her doctor’s advice and loses around 15 Kg within 6 months.” In the other two vignettes, the doctors recommend starting a diet and regular exercise program or undergoing bariatric surgery** to lose weight (it was also explained that **bariatric surgery intervenes on the stomach through specific procedures to achieve weight loss). The weight was expressed in Kg and the height in m, as they are the units of measurement in the European system.

*Responsibility for weight loss* was measured with a single item, “*How much is the patient personally responsible for losing weight after the visit with the doctor*?” (as done in Mattingly et al., 2009; Vartanian and Fardouly, 2014), rated using a 5-point scale (1 = not at all responsible and 5 = totally responsible).

*Judgments toward the doctor and treatment*. Participants responded to six questions assessing their perception of the doctor and the proposed treatment. Trust in the physician was assessed with a single item (“*How much do you trust the doctor in the story*?”). Perceived competence of the doctor was measured using three items: “*How likely would you be to visit this doctor if you had an obesity problem?*,” “*How likely would you be to recommend this doctor to an obese relative or friend?*,” and “*How competent does the doctor in the story appear*?.” These items were averaged to form a composite index of doctor evaluation, with higher scores indicating greater endorsement of the doctor and treatment (*ω* = 0.93, rs > 0.81).

Treatment endorsement was assessed by two items: “*How likely would you be to consider starting the treatment proposed by the doctor if you were obese?*,” and “*How likely would you be to recommend the treatment proposed by the doctor to an obese relative or friend?*.” These items were averaged to create a treatment evaluation index, with higher scores indicating stronger endorsement of the treatment. All items were rated on a 5-point Likert scale (1 = not at all and 5 = totally agree).

## Results

The pre-test, conducted to check randomization, showed that participants across the three conditions of the experimental manipulation were similar in their socio-demographic variables (see Tables 1, 2). Willpower did not correlate with age, BMI, general health status, and self-evaluation of the weight (*p* > 0.05). Among the socio-demographic variables, only gender was associated with lack of willpower, *t*(151) = 4.57, *p* < 0.001, with men showing higher lack of willpower (*M* = 3.6, SD = 0.12) than women (*M* = 2.9, SD = 0.82).

**Table 1:** Frequency distributions and results of the chi-square.

	Variables	Diet exercise (*n*)	Bariatric surgery (*n*)	Weight-loss drugs (*n*)	χ^2^ (df)
Gender	Males	11	12	12	(2) = .18
	Females	40	42	36	
In a relationship	Yes	24	29	31	(2) = 3.11
	No	27	25	17	
Education	No title/compulsory school	4	3	1	(4) = 3.1
	Diploma	24	30	22	
	University degree	23	21	25	
Source of income	Yes	22	19	17	(2) = .88
	No	29	35	31	
Chronic illness	Yes	9	5	5	(2) = 1.9
	No	42	49	43	
Diet in the past	Yes	29	20	32	(2) = 9.42**^a^
	No	22	34	16	
Familiarity with obesity	Yes	22	24	26	(2) = 1.4
	No	29	30	22	

** = *p* < 0.01; ^a^=standardized residuals were not significant.

**Table 2:** Frequency distribution and results of the anovas.

	Diet exercise (*M*)	Bariatric surgery (*M*)	Weight-loss drugs (*M*)	F(df df)
Age	29.9	28.5	29.5	(2 80) = .28
BMI	25.3	24.7	25.1	(2 179) = .08
General health status	3.59	3.74	13.81	(2 150) = .92
Self-evaluation of weight	3.47	3.37	3.56	(2 150) = .57

**Figure 1:**
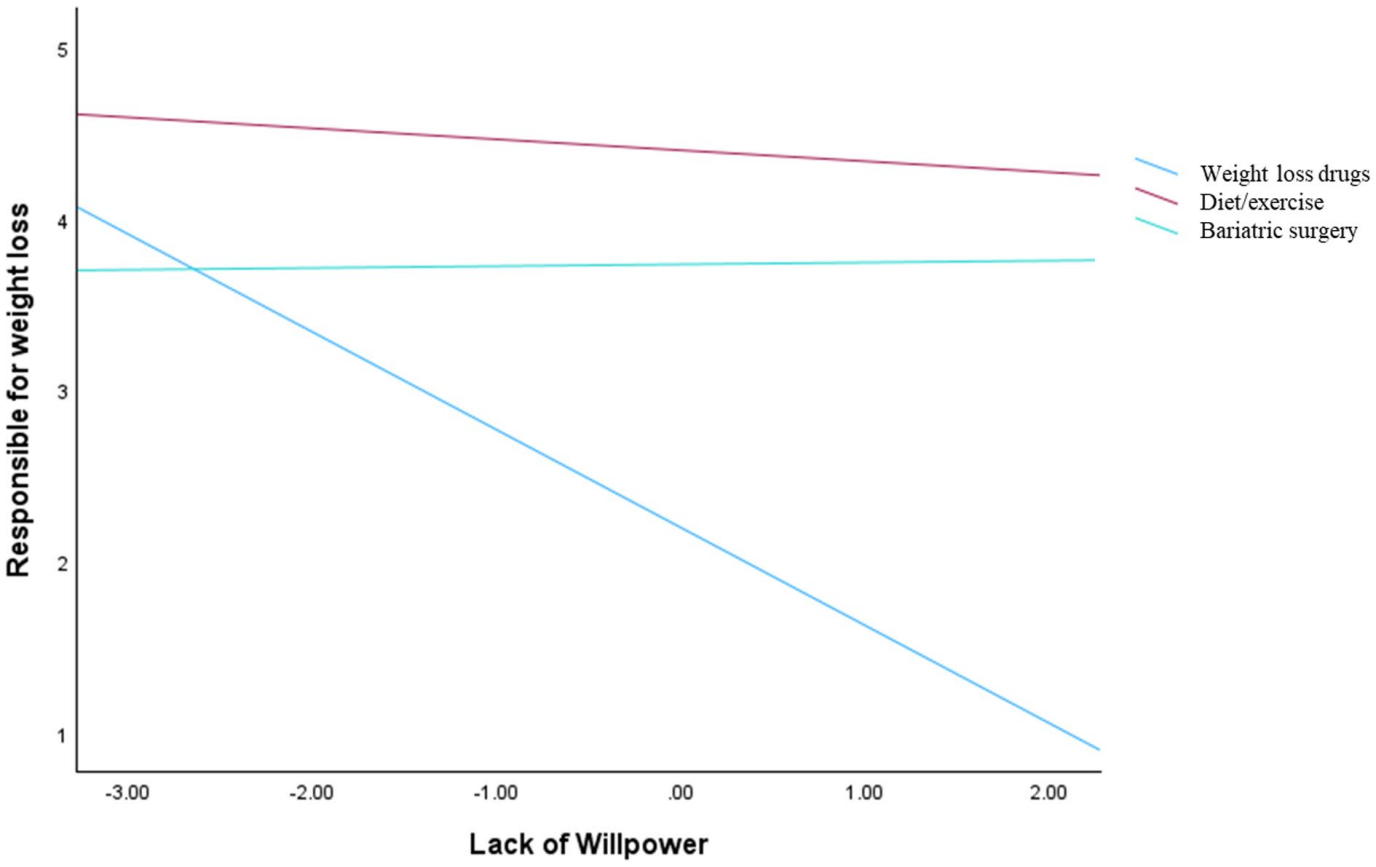
Interaction effect between lack of willpower and manipulation conditions on responsibility for weight loss.

A moderation analysis was conducted to examine whether the treatment type moderated the relationship between perceived lack of willpower and responsibility for weight loss, controlling for gender, BMI, age, and dieting status. The overall model was significant, *F*(8, 143) = 23.46, *p* < 0.001, explaining 56.75% of the variance (*R*^2^ = 0.5675). Consistent with HP1, a significant effect of perceived lack of willpower emerged in the reference group (drug condition), such that higher perceived lack of willpower was associated with lower responsibility for weight loss (*b* = −0.59, SE = 0.14, *p* < 0.001). Supporting HP2, a significant main effect of the treatment type was observed: compared to the drug condition, participants attributed higher responsibility to individuals undergoing surgery (*b* = 2.26, SE = 0.17, *p* < 0.001) and those engaging in diet and exercise (*b* = 1.68, SE = 0.17, *p* < 0.001), at the mean level of perceived lack of willpower. In line with HP3, the interaction between the perceived lack of willpower and treatment type was significant, Δ*R*^2^ = 0.0302, *F*(2, 143) = 5.00, *p* = 0.008. Simple slope analyses (Figure 1) revealed that perceived lack of willpower was negatively associated with responsibility for weight loss in the drug condition (*b* = −0.59, SE = 0.14, *p* < 0.001), but not in the surgery (*b* = −0.09, SE = 0.15, *p* = 0.541) or diet/exercise conditions (*b* = −0.04, SE = 0.15, *p* = 0.811). None of the covariates showed significant effects.

**Figure 2:**
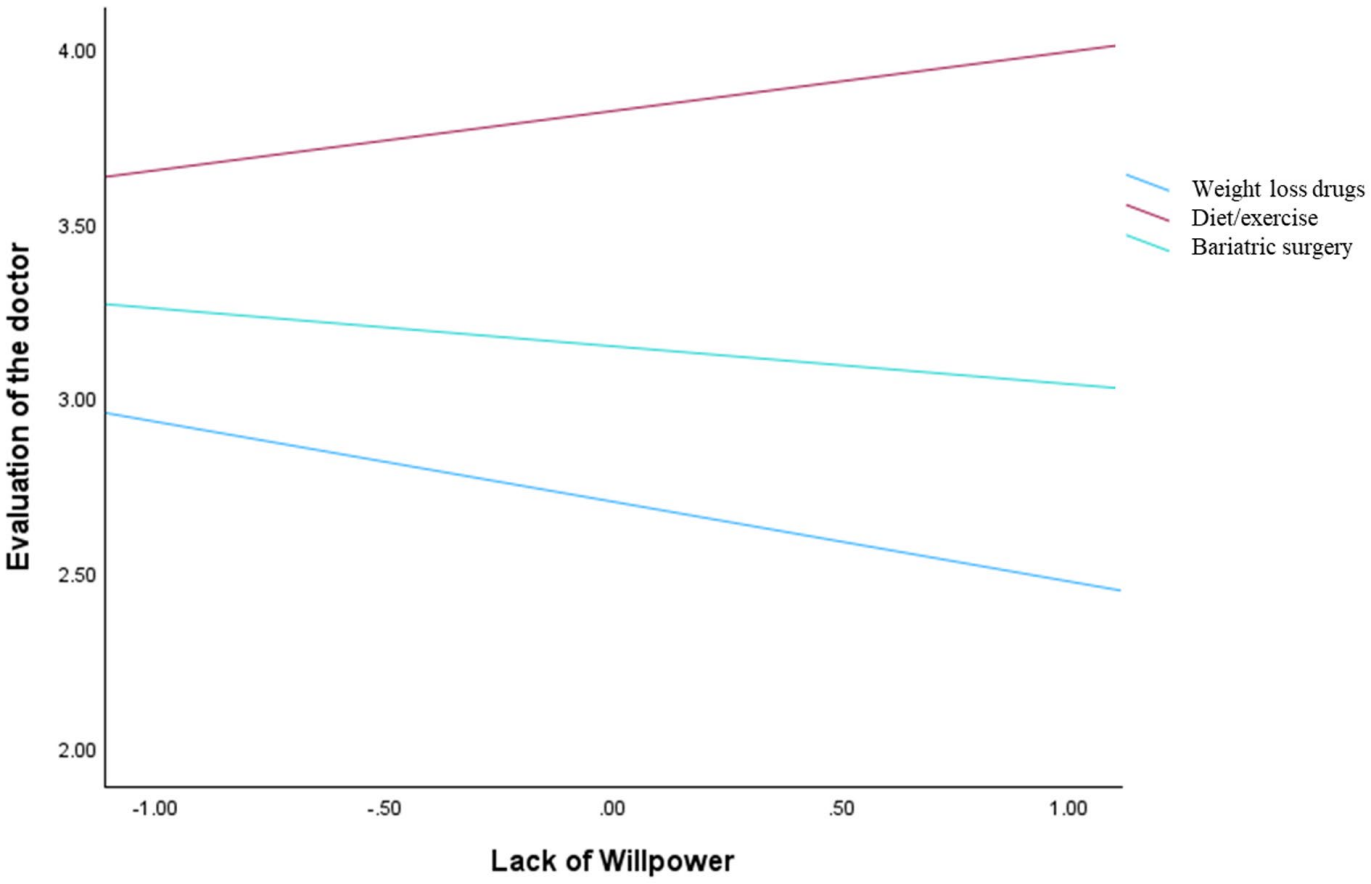
Interaction effect between lack of willpower and manipulation conditions on evaluations of the doctor.

A moderation analysis was conducted to examine whether the treatment type moderated the relationship between perceived lack of willpower and evaluations of the doctor, while controlling for gender and BMI. The overall model was significant, *F*(6, 146) = 6.23, *p* < 0.001, explaining 20.38% of the variance (*R*^2^ = 0.2038). Consistent with HP4, the main effect of perceived lack of willpower was not significant (*b* = −0.23, SE = 0.15, *p* = 0.138). Significant main effects of treatment type emerged, supporting HP5: compared to the drug condition, evaluations of the doctor were higher in both the surgery (*b* = 1.12, SE = 0.19, *p* < 0.001) and diet/exercise conditions (*b* = 0.44, SE = 0.19, *p* = 0.022), at the mean level of perceived lack of willpower. Finally, the interaction between perceived lack of willpower and treatment type was not significant, Δ*R*^2^ = 0.0179, *F*(2, 146) = 1.64, *p* = 0.197 (Figure 2).

**Figure 3:**
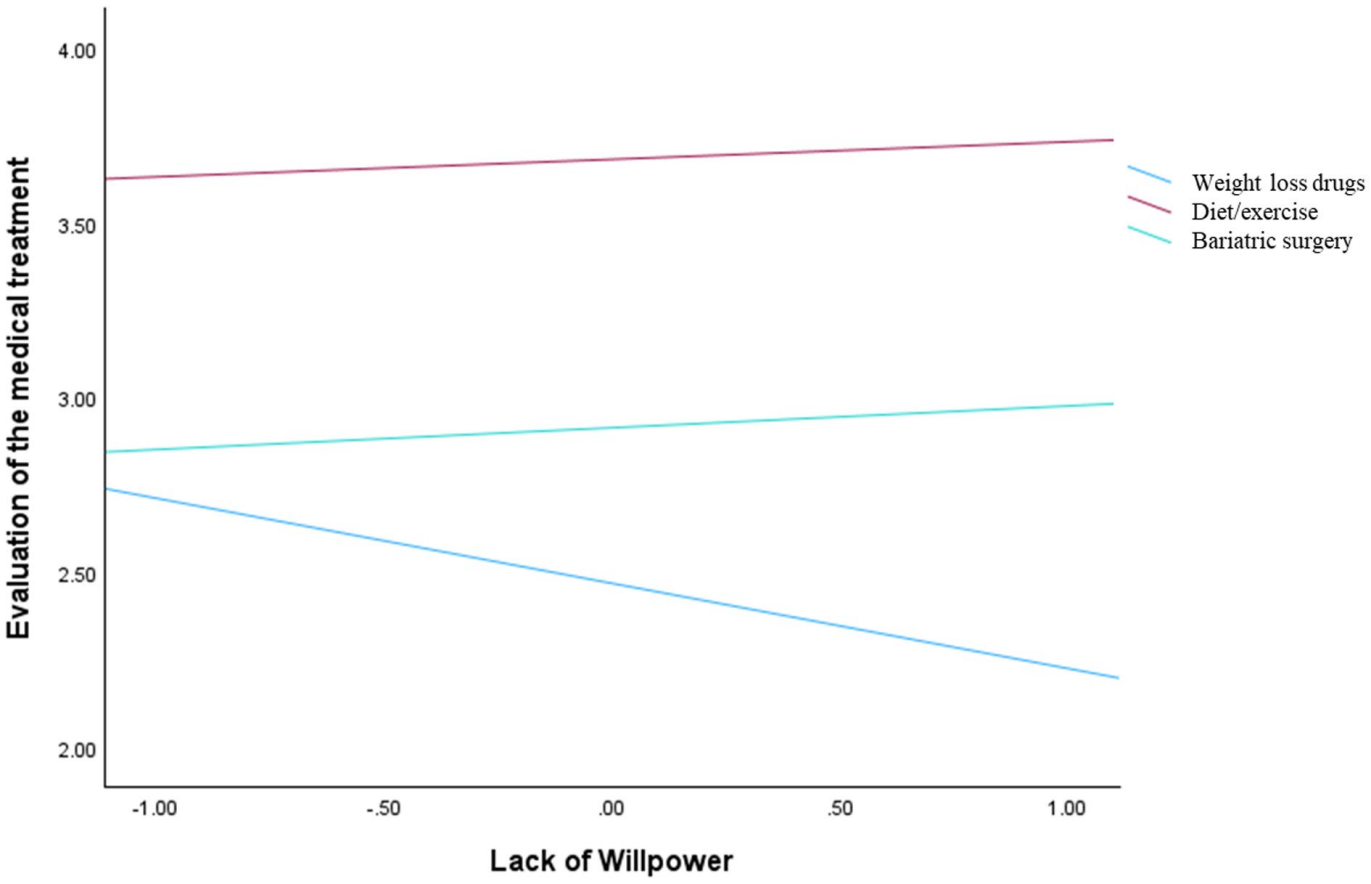
Interaction effect between lack of willpower and manipulation conditions on the evaluations of the medical treatment.

**Figure 4:**
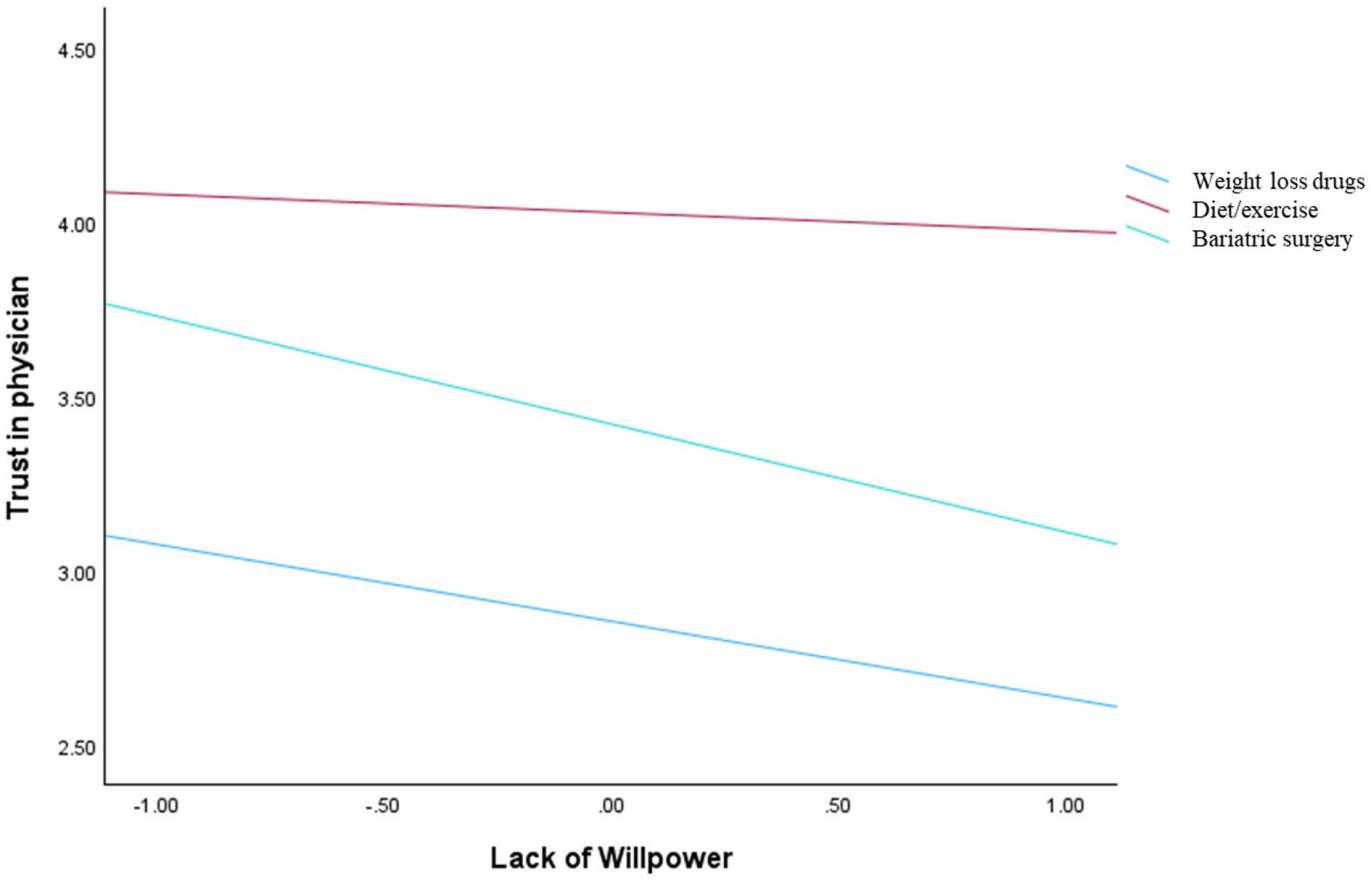
Interaction effect between lack of willpower and manipulation conditions on trust in the physician.

A similar pattern emerged for both the evaluation of treatment and trust in the physician. The overall model was significant for evaluation, *F*(6, 146) = 5.94, *p* < 0.001 (*R*^2^ = 0.1963), and for trust, *F*(6, 146) = 6.06, *p* < 0.001 (*R*^2^ = 0.1995). The main effects of perceived lack of willpower were not significant (*b* = −0.24, SE = 0.17, *p* = 0.145, and *b* = −0.22, SE = 0.16, *p* = 0.17, respectively) as well as the interaction terms (Figures 3, 4).

## Discussion

The present study investigated how beliefs about personal responsibility for weight loss and perceptions of healthcare professionals are shaped by both the type of obesity treatment and the perceived willpower attributed to individuals who live with obesity. When the dependent variable was the individual’s perceptions of weight loss control, both the lack of willpower and treatment type had significant effects, qualified by a significant interaction between the two. Specifically, the effect of perceived willpower emerged primarily in the drug condition, where participants attributed less responsibility for weight loss when willpower was perceived as lacking, whereas this relationship was not observed in the surgery and diet/exercise conditions.

These findings extend previous research on attribution processes in obesity, showing that attributing obesity to a lack of willpower leads to greater blame and harsher judgments toward individuals with obesity (Crandall, 1994; Weiner et al., 1988). First, these results suggest that such attributions may also shape perceptions of responsibility for change. While individuals may be blamed for their condition, they may simultaneously be perceived as less responsible for successful weight loss. Second, this specific influence becomes more evident when weight loss is achieved through treatments perceived as more externally driven, as these outcomes are less likely to be attributed to an individual’s effort. In this sense, although responsibility for weight loss is conceptually distinct from blame, it may still carry evaluative implications. Lower responsibility for weight loss may reflect a tendency to discount the individual’s agency and effort, thereby indirectly signaling negative judgments about the person. This pattern highlights potential asymmetry in attribution processes, whereby individuals may be held responsible for the onset of obesity but not credited for its resolution. Importantly, the measure of lack of willpower reflects both causal attributions and evaluative, potentially stigmatizing beliefs, which may partly account for its association with responsibility judgments.

Furthermore, individuals undergoing drug-based treatment were seen as having less control over their weight loss compared to those following diet and exercise. This may be linked to the perception that weight loss drugs are less effortful and are associated with lower attributions of personal agency (Puhl and Heuer, 2009; Weiner et al., 1988). The observed difference in perceived control between drug-based and lifestyle-based weight loss mirrors findings from research on treatment acceptability and stigma. Physicians commonly believe that patients with obesity lack motivation to make effortful lifestyle changes and that they are not compliant with treatments that require effort (Brown, 2006; Campbell et al., 2000; Campbell and Crawford, 2000; Thuan and Avignon, 2005). The reduced sense of control attributed to individuals undergoing drug-based treatments also reflects existing biases identified in the literature. Research on mental health, for example, has noted that pharmacological interventions for depression and anxiety are often perceived as less effortful or more “passive” compared to psychotherapy or lifestyle-based approaches (Deacon and Baird, 2009), and are then perceived as less legitimate (Sirey et al., 2001).

When the dependent variables concerned the doctor’s judgment (i.e., trust, perceived competence, and treatment endorsement), perceived lack of willpower did not significantly influence these evaluations, nor did it interact with treatment type. This indicates that attributional judgments about individuals with obesity rely on dispositional inference processes, whereas evaluations of professionals are driven by heuristic cues related to action and intervention (Chaiken, 1980; Petty and Cacioppo, 1986). Across all outcomes, pharmacological treatments were consistently associated with lower evaluations compared to surgery and diet/exercise. In this sense, treatment type functions as a signal that informs judgments of expertise and legitimacy, independently of beliefs about the patient’s willpower. In line with research on effort-based heuristics (Olivola and Shafir, 2013), treatments perceived as requiring greater effort and involvement may be interpreted as more legitimate and professionally appropriate. Medications are often seen as requiring less personal effort (Andreassen et al., 2024; Post and Persky, 2024), and patients may perceive that doctors are simply prescribing a quick fix rather than offering a comprehensive, professional solution. Treatments that are seen as requiring greater effort, involvement, and expertise, such as surgery and lifestyle-based interventions, may lead to more favorable evaluations of the doctor. In contrast, pharmacological approaches may be perceived as less demanding or less effortful, resulting in lower perceived competence, trust, and endorsement.

The implication for clinical practice is that healthcare providers should be aware of how perceptions of treatment options can unconsciously influence both their own recommendations and patients’ acceptance of care. Recognizing and actively addressing these biases is essential to ensure that all treatment options, whether behavioral, surgical, or pharmacological, are presented as equally valid components of a comprehensive, personalized care plan. By fostering a non-judgmental therapeutic environment, clinicians can promote greater trust, improve adherence, and support more equitable access to appropriate interventions. For individuals with obesity or overweight, a patient-centered approach may offer personalized communication about the broad range of options, emphasizing that medical interventions are not signs of failure but tools to support their health when other approaches have proven insufficient. Ultimately, this approach can foster more open, trust-based doctor–patient relationships, as patients feel their concerns, experiences, and values are respected in the treatment process.

While the study provides insights into perceptions of obesity and weight loss, several limitations should be noted. First, the sample sizes, though adequate for the proposed analyses, may only partially represent the broader population. Although young people are the group most exposed to negative online communication about weight loss drugs, the sample was relatively young, predominantly female, and recruited through convenience sampling within the Swiss context. These characteristics may limit the generalizability of the findings. Second, relying on self-reported measures (e.g., willpower) and single-item measures (e.g., trust and responsibility for weight loss) introduces potential biases, such as social desirability. Future research could incorporate objective measures, such as implicit attitude tests, to strengthen our findings. Third, the experimental manipulation of weight loss methods (drugs, surgery, and diet/exercise) was based on vignettes, which may only partially reflect the complexity of provider–patient interactions (e.g., non-verbal cues) and real-world decision-making. Future studies should also use longitudinal designs to measure judgments before and after exposure to real-world health campaigns or medical treatments, including GLP-1 receptor antagonists, given their clinical relevance, and consider possible cultural differences. A further limitation concerns the role of moralization. Although the study was theoretically informed by perspectives on the moralization of health, moralization was not directly operationalized as a central construct. Future research should include explicit moralization measures to assess its role in shaping responsibility attributions and evaluations of weight loss treatments.

## Conclusion

Given the increasing reliance on pharmacological and surgical interventions for weight management, future research should examine how these evolving treatment landscapes influence public and clinical perceptions of obesity. Moreover, since in actual clinical situations a combination of medication, lifestyle interventions, and sometimes surgery is used, future studies should consider these medical approaches when designing future development. Health campaigns, for instance, may benefit from reframing obesity as a complex medical condition shaped by biological, psychological, and environmental factors, rather than as a result of individual failure. Such reframing could help reduce weight-related stigma and increase openness to diverse, evidence-based treatment options. Equally important is exploring how healthcare professionals can communicate treatment recommendations in ways that preserve patients’ dignity and trust. Training clinicians to present all treatment options, whether behavioral, surgical, or pharmacological, without implicit moral judgments may foster stronger therapeutic relationships and improve adherence.

## Data Availability

The raw data supporting the conclusions of this article will be made available by the authors, without undue reservation.
